# Impact of Neoadjuvant Chemotherapy on Surgical Outcomes and Conversion to Node-Negativity in Invasive Lobular Breast Cancer: Analysis of Molecularly High-Risk Tumors by Histologic Subtype on the I-SPY2 Clinical Trial

**DOI:** 10.1245/s10434-025-17862-0

**Published:** 2025-07-24

**Authors:** Rita A. Mukhtar, Katrina Dimitroff, Christina Yau, A. Jo Chien, Eileen P. Connolly, Marissa Howard-McNatt, Roshni Rao, Velle Ladores, Mehra Golshan, Candice A. Sauder, Kamran Ahmed, Rachael Lancaster, Jana Fox, Lily Gutnik, M. Catherine Lee, Julia Tchou, Nicolas Prionas, Cletus A. Arciero, Chantal Reyna, Henry Kuerer, Kayla Switalla, Neil Taunk, Todd M. Tuttle, Meena S. Moran, Lauren M. Postlewait, Jane Perlmutter, Angela DeMichele, Douglas Yee, Nola Hylton, W. Fraser Symmans, Hope S. Rugo, Rebecca Shatsky, Claudine Isaacs, Laura J. Esserman, Laura van’t Veer, Judy C. Boughey

**Affiliations:** 1 UC San Francisco, 1825 4th st, San Francisco, CA 94158 USA; 2 Columbia University, New York, USA; 3 Wake Forest, Winston-Salem, USA; 4 Yale, New Haven, USA; 5 University of California, Davis, Davis, USA; 6 H. Lee Moffitt Cancer Center, Tampa, USA; 7 University of Alabama Birmingham, Birmingham, USA; 8 Montefiore Medical Center, Bronx, USA; 9 University of Pennsylvania, Philadelphia, USA; 10 Emory, Atlanta, USA; 11 Loyola University, Chicago, USA; 12 MD Anderson Cancer Center, Houston, USA; 13 University of Minnesota, Minneapolis, USA; 14 Gemini Group, Ann Arbor, MI USA; 15 UC San Diego, San Diego, USA; 16 Georgetown University, Washington, USA; 17 Mayo Clinic, Rochester, USA

**Keywords:** Breast cancer, Neoadjuvant chemotherapy, Nodal response, Lobular carcinoma

## Abstract

**Background:**

Invasive lobular carcinoma (ILC) has lower response rates to neoadjuvant chemotherapy (NAC) than invasive ductal carcinoma. While ILC often has low-risk biology, there is a high-risk subset within this heterogeneous tumor type. We compared surgical treatment and response rates by histology in I-SPY2, a multicenter NAC trial.

**Methods:**

We evaluated 1329 patients with stage II–III breast cancer and high-risk 70-gene assay. Patients with classic, pleomorphic, or mixed lobular/ductal histology were included in the lobular cohort. We evaluated rates of mastectomy, positive margins, axillary dissection, and conversion from clinical node-positive (cN+) to pathologic node-negative (ypN−) status after NAC.

**Results:**

Overall, 124 patients (9.3%) had lobular histology, with 69% being hormone receptor-positive/human epidermal growth factor receptor 2-negative (HR+/HER2−). There was no difference in mastectomy rate (57.2% for lobular vs. 55.8% for non-lobular). The ILC cohort had more positive margins after lumpectomy than the non-ILC cohort (21.2% vs. 7.9%; *p* = 0.023). Within cN0 cases, axillary dissection was significantly more common among the lobular cases (24.1% vs. 14.0%; *p* = 0.039). Conversion from cN+ to ypN0 did not differ statistically between lobular and non-lobular cases (40.9% vs. 51.2%; *p* = 0.11). The nodal conversion rate among cN+lobular tumors was 30.6% in HR+/HER2−, 72.7% in HER2+, and 66.7% in triple-negative cases.

**Conclusions:**

These data demonstrate the challenges of surgical management for ILC but hold promise that molecular classification can improve treatment selection. While high genomic risk is generally less common among ILC, our findings suggest that gene expression assays in cN+ILC patients can identify a subset who may benefit from NAC.

**Supplementary Information:**

The online version contains supplementary material available at 10.1245/s10434-025-17862-0.

Neoadjuvant chemotherapy (NAC) in breast cancer allows for downstaging of disease, which can improve surgical outcomes, provide prognostic information, and guide adjuvant therapy.^1–3^ A clear benefit of NAC is the increase in rates of breast conservation therapy, facilitated by a reduction in the size of the primary tumor in the breast. NAC can also lead to nodal downstaging or eradication of tumor involvement in axillary lymph nodes. While pathologic complete response (pCR) in the breast and axillary nodes may occur together, having a nodal pCR even in the absence of breast pCR is still a favorable prognostic indicator.^4^ Since targeted axillary dissection (TAD) can now be offered to clinical node-positive (cN+) patients who have nodal response to therapy, NAC may be especially beneficial for patients with nodal involvement who may then avoid axillary lymph node dissection (ALND).^5^ Such de-escalation in the surgical treatment of the axilla can reduce the risk of complications, including lymphedema.

However, not all patients have the same likelihood of response to NAC. In particular, numerous studies have shown that patients with invasive lobular carcinoma (ILC) have lower response rates to NAC compared with those with invasive ductal carcinoma (IDC) or carcinoma of no special type (NST).^6,7^ ILC is the second most common histologic type of breast cancer, representing 10–15% of all breast cancer cases. Patients with ILC have lower rates of overall pCR and higher rates of mastectomy and ALND than those with IDC.^8,9^

These lower response rates to NAC may be due to the high prevalence of hormone receptor-positive (HR+) and human epidermal growth factor 2-negative (HER2−) subtype observed among lobular tumors, combined with generally lower proliferation rates and lower incidence of high-grade disease.^10^ Because of this, some have suggested that patients with ILC should not receive NAC and should instead be treated with upfront surgery.^11,12^ However, heterogeneity exists in ILC, and a subset of even HR+/HER2− ILC tumors have molecularly high-risk disease, as measured by genomic assays such as the 70-gene assay (Mammaprint) and the 21-gene recurrence score (Oncotype Recurrence Score).^13–17^

Determining which patients with ILC will benefit from chemotherapy remains a major clinical challenge. This is especially important because patients with ILC present at higher stages than patients with non-lobular histology, likely due to decreased sensitivity of screening mammography for ILC.^18–20^ Identifying the subset of ILC patients who are predicted to have response to NAC would improve our ability to appropriately select therapy for these patients.

In this study, we evaluated patients who were treated with NAC on the I-SPY2 trial (ClinicalTrials.gov identifier: NCT01042379) and compared surgical outcomes and nodal response rates by histologic subtype. I-SPY2 is a prospective, randomized, multicenter, adaptive platform trial for patients with molecularly high-risk breast cancer. Our primary goal was to evaluate whether lobular histology was associated with different surgical treatment compared with non-lobular histology in the context of a randomized NAC trial. Our secondary goal was to determine the rate of nodal pCR in this unique cohort of Mammaprint high-risk lobular tumors, stratified by tumor receptor subtype.

## Methods

**Fig. 1:**
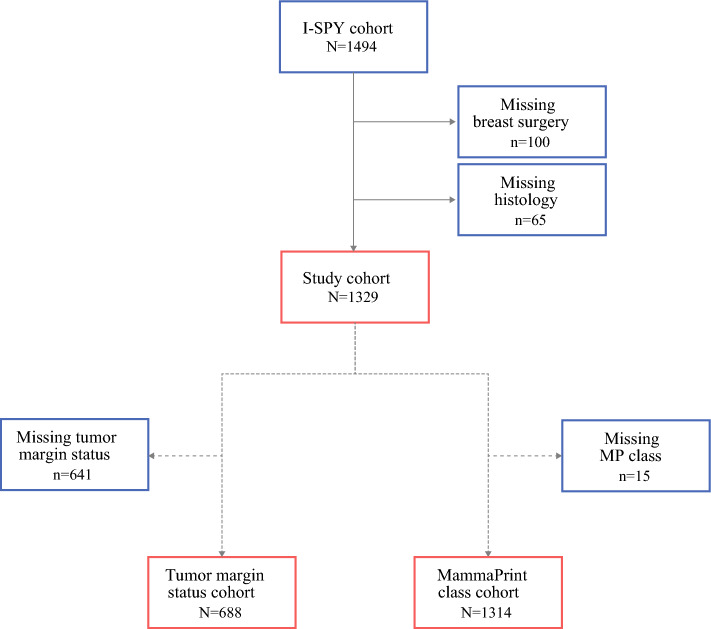
CONSORT diagram. The total study population consisted of 1329 patients with Mammaprint high-risk breast cancer who completed neoadjuvant therapy and surgery and had histology subtype data available. Of these patients, 1314 had Mammaprint class (H1 vs. H2) data available and 688 had tumor margin status available for analysis. *CONSORT* Consolidated Standards of Reporting Trials

We included all patients who completed therapy on the I-SPY2 trial between the years 2010 and 2021 at 19 sites across the United States. Patients missing breast surgery and histology information were excluded (Fig. 1). Mammaprint scores were obtained from pretreatment breast core needle biopsy specimens and were further stratified as high 1 (H1) or high 2 (H2) ^21^ Mammaprint scores between 0 and − 0.56 constitute H1, and scores between − 0.57 and − 1.0 constitute H2. Mammaprint H2 tumors are characterized by higher expression of proliferation-related, DNA repair-related, and immune-related genes, and are considered to have higher-risk biology.^22^ Estrogen (ER) and progesterone receptor (PR) status were determined by immunohistochemistry, with ≥5% staining being considered positive. Of note, patients on I-SPY2 underwent randomization to systemic therapy arms designed specifically for tumor molecular subtype, including ER status; use of adjuvant endocrine therapy for patients with ER low-positive tumors was not dictated by the trial. HER2 status was determined by immunohistochemistry (3+ considered positive) or by fluorescence in situ hybridization. Tumor receptor subtype was grouped as HR+/HER2−, HR−/HER2− (triple negative), or HER2+. Tumor grade was determined using the Scarff–Bloom–Richardson system.

Eligibility for I-SPY2 requires clinical tumor size ≥ 2.5 cm and either triple-negative, HER2+, or HR+/HER2− subtype with high-risk Mammaprint score. Patients on I-SPY2 are randomized to multiple arms of novel NAC agents, stratified by tumor receptor subtype and molecular subtype (Mammaprint H1 vs. H2). Before and during NAC, patients are monitored with serial breast magnetic resonance imaging (MRI) with surgical treatment following completion of systemic therapy. Type of surgery (breast conservation vs. mastectomy, and sentinel lymph node [SLN] surgery vs. ALND) is not dictated by the study but rather selected by the treating surgeon based on clinical features and patient preference. Surgical specimens are evaluated per standard clinical protocols, and residual cancer burden is calculated by trained pathologists at each participating site.

Type of surgical treatment and margin status were obtained from case report forms from participating sites and chart review of operative and pathology reports. When multiple surgical procedures were performed, the final oncologic operation was considered the definitive procedure (i.e., a patient who underwent lumpectomy followed by mastectomy was categorized in the mastectomy group). Surgical margins were considered positive if there was ‘ink on tumor’.

T category was grouped as T1/2 versus T3/4 at both pretreatment (cT) and post-treatment (from surgical specimen, ypT); those with complete pathologic response in the breast were categorized as ypTis or ypT0. We considered both clinical nodal status (prior to NAC) and pathologic nodal status (post-treatment). Nodal status was classified as positive or negative, with cN+ patients having needle biopsy-proven lymph node involvement prior to NAC, and other patients being considered clinical node-negative (cN0). Pathologic nodal status was similarly classified as positive at axillary surgery (ypN+) or negative (ypN0).

We defined two study cohorts—the ILC and non-ILC cohorts. Patients with classic lobular, pleomorphic lobular, or mixed lobular/ductal tumors were included in the ILC cohort. Patients with all other tumor histologies were grouped in the non-ILC cohort; the majority of these were IDC (98.3%). We used Pearson’s Chi-square test or Fisher's exact test to compare clinical features between the ILC and non-ILC groups and used the Wilcoxon rank-sum test for continuous variables. Differences in type of breast surgery (mastectomy vs. breast-conserving surgery), type of axillary surgery (SLN vs. ALND), and rates of conversion of nodal status (cN+ to ypN0) were compared between groups. Patients who were cN+ but pathologically node-negative (ypN0) were considered to have had a nodal response to NAC (nodal pCR).

Further subset analyses were performed within the ILC cohort and between the ILC and non-ILC cohorts stratified by tumor receptor subtype and Mammaprint class (H1 or H2). Statistical analyses were performed using R version 4.3.1 (R Foundation for Statistical Computing, Vienna, Austria). All statistical tests were two sided and a *p*-value <0.05 was considered significant.

## Results

### Overall Study Cohort

**Fig. 2:**
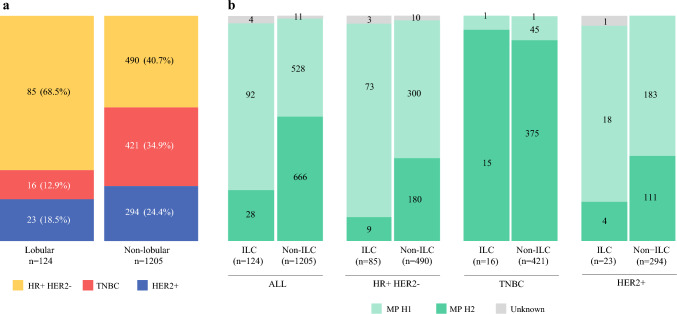
(**a**) Differences in the distribution of tumor receptor subtypes in lobular versus non-lobular cases. (**b**) Distribution of Mammaprint H1 versus H2 cases, stratified by histologic subtype (lobular vs. non-lobular) and tumor receptor subtype. *HR* hormone receptor, *HER2* human epidermal growth factor receptor 2, *TNBC* triple-negative breast cancer, *ILC* invasive lobular carcinoma, *MP* Mammaprint, *H1* high 1, *H2* high 2

We included 1329 patients with Mammaprint high-risk breast cancer who completed assigned NAC on the I-SPY2 trial and underwent surgical treatment (Table 1). The mean age at screening was 48.3 years (range 19–80). Of these patients, 124 (9.3%) had lobular histology and 1205 (90.7%) had non-lobular histology. The lobular group consisted of 77 (62.1%) patients with mixed ILC/IDC, 30 (24.2%) with classic ILC, and 17 (13.7%) with pleomorphic ILC. Compared with the non-lobular group, those with ILC were significantly more likely to have HR+/HER2− tumors, lower-grade disease, and older age (Table 1, Fig. 2). Clinical stage at presentation did not differ significantly between groups. Among the lobular cohort, 37.1% had clinical T3/4 tumors, compared with 29.7% of the non-lobular cohort. Similar proportions were cN+ in each group (53.2% in the lobular group and 52.5% in the non-lobular group). Tumor grade was only available in 69.9% of the entire study cohort but there was a significantly lower proportion of grade 3 tumors among the lobular cohort compared with the non-lobular cohort (30.9% vs. 73.7%; *p* < 0.001).

**Table 1:** Patient and pretreatment clinical characteristics overall and by histology

Characteristic	Total [*N* = 1329]	Lobular [*n* = 124]	Non-lobular [*n* = 1205]	*p*-Value^a^
Age, years [mean (SD)]	48.18 (11.09)	50.85 (9.55)	47.91 (11.21)	**0.002**
Race				0.7
White	1054/1329 (79.3)	104/124 (83.9)	950/1205 (78.8)	
Black	148/1329 (11.1)	12/124 (9.7)	136/1205 (11.3)	
Asian	106/1329 (8.0)	7/124 (5.6)	99/1205 (8.2)	
Other	21/1329 (1.6)	1/124 (0.8)	20/1205 (1.7)	
Ethnicity				0.14
Non-Hispanic or Latino	1173/1329 (88.3)	116/124 (93.5)	1057/1205 (87.7)	
Hispanic or Latino	155/1329 (11.1)	8/124 (6.5)	147/1205 (12.2)	
Unknown	1/1329 (0.1)	0/124 (0)	1/1205 (<0.1)	
Baseline clinical T category				0.089
T1-T2	925/1329 (69.6)	78/125 (62.9)	847/1205 (70.3)	
T3-4	404/1329 (30.4)	46/124 (37.1)	358/1205 (29.7)	
Baseline clinical node status				0.9
Negative	630/1329 (47.4)	58/124 (46.8)	572/1205 (47.5)	
Positive	699/1329 (52.6)	66/124 (53.2)	633/1205 (52.5)	
Pathologic T category after NAC				**<0.001**
T0/Tis	529/1326 (39.9)	32/124 (25.8)	497/1202 (41.3)	
T1-T2	665/1326 (50.2)	71/124 (57.3)	594/1202 (49.4)	
T3-T4	132/1326 (10.0)	21/124 (16.9)	111/1202 (9.2)	
Missing	3	0	3	
Pathologic node status after NAC				**0.031**
Negative	887/1329 (66.7)	72/124 (58.1)	815/1205 (67.6)	
Positive	442/1329 (33.3)	52/124 (41.9)	390/1205 (32.4)	
Tumor receptor subtype				**<0.001**
HR+/HER2−	575/1329 (43.3)	85/124 (68.5)	490/1205 (40.7)	
TNBC	437/1329 (32.9)	16/124 (12.9)	421/1205 (34.9)	
HER2+	317/1329 (23.9)	23/124 (18.5)	294/1205 (24.4)	
Tumor grade				**<0.001**
I/II	279/929 (30.0)	56/81 (69.1)	223/848 (26.3)	
III	650/929 (70.0)	25/81 (30.9)	625/848 (73.7)	
Missing	400	43	357	
MammaPrint class				**<0.001**
H1	620/1314 (47.2)	92/120 (76.7)	528/1194 (44.2)	
H2	694/1314 (52.8)	28/120 (23.3)	666/1194 (55.8)	
Missing	15	4	11	

Data are expressed as *n*/*N* (%) unless otherwise specified

*SD* standard deviation, *NAC* neoadjuvant chemotherapy, *HR* hormone receptor, *HER2* human epidermal growth factor receptor 2, *TNBC* triple-negative breast cancer, *H1* high 1, *H2* high 2

^a^Wilcoxon rank-sum test, Fisher’s exact test, Pearson’s Chi-square test

The distribution of Mammaprint class H1 and H2 differed significantly by histologic group, with fewer H2 tumors in the ILC group than in the non-ILC group (23.3% vs. 55.8%; *p* < 0.001). This was driven by patients with HR+/HER2- tumors, with 11.0% H2 cases in the ILC group versus 37.5% H2 cases in the non-ILC group among this tumor receptor subtype (*p* < 0.001). There was no difference in the proportion of H2 tumors by histologic subtype among triple-negative or HER2+ cases. Among the triple-negative lobular tumors (*n* = 16), 93.8% were H2, compared with 89.3% among the 420 triple-negative non-lobular tumors. Among the HER2+ lobular tumors (*n* = 23), 18.2% were H2, compared with 37.8% among the 316 HER2+ non-lobular tumors.

### Breast Surgery and Positive Margin Rates

Overall, 585 patients (44.0%) underwent BCS as their final surgery, and 744 patients (56.0%) underwent mastectomy. There was no difference in the final rate of BCS by histology, with 53 patients (42.7%) undergoing BCS in the lobular group and 532 patients (44.1%) undergoing BCS in the non-lobular group (Table 2). This remained true within histology groups stratified by tumor receptor subtype (electronic supplementary material [ESM] Table S1). Notably, ypT category was larger in the lobular group. For lobular cases, 16.9% had ypT3/4 tumors at surgery compared with 9.2% of non-ILC cases (*p* < 0.001). Additionally, the relationship between cT and ypT status differed by histologic group; among those with cT1/2 disease, upstaging to ypT3/4 category occurred in 12.8% of the lobular cohort compared with 4.4% of the non-lobular cohort (*p* < 0.001).

**Table 2:** Surgical treatment by histologic group

	Total	Lobular	Non-lobular	*p*-Value^a^
Characteristic, *n*	1329	124	1205	
Type of breast surgery				0.8
BCS	585/1329 (44.0)	53/124 (42.7)	532/1205 (44.1)	
Mastectomy	744/1329 (56.0)	71/124 (57.3)	673/1205 (55.9)	
Tumor margin status				
Overall, *n*	688	84	604	**0.005**
Negative	648/688 (94.2)	73/84 (86.9)	575/604 (95.2)	
Positive	40/688 (5.8)	11/84 (13.1)	29/604 (4.8)	
Missing	641	40	601	
Positive margin rate after BCS [*n* = 298]	28/298 (9.4)	7/33 (21.2)	21/265 (7.9)	**0.023**
Positive margin rate after mastectomy [*n* = 390]	12/390 (3.1)	4/51 (7.8)	8 / 339 (2.4)	0.058
Axillary surgery				0.2
SLN	805/1329 (60.6)	69/124 (55.6)	736/1205 (61.1)	
ALND	524/1329 (39.4)	55/124 (44.4)	469/1205 (38.9)	
Mean total nodes removed [mean (SD)]	8.64 (8.49)	9.64 (8.94)	8.53 (8.44)	0.14
Mean positive nodes [mean (SD)]	1.32 (3.33)	2.26 (4.77)	1.23 (3.13)	**0.005**
Axillary surgery in cN0 cases [*n* = 630]				0.039
SLN	536/630 (85.1)	44/58 (75.9)	492/572 (86.0)	
ALND	94/630 (14.9)	14/58 (24.1)	80/572 (14.0)	
Number of total nodes removed in cN0 cases [mean (SD)]	**5.11 (6.23)**	**7.43 (9.13)**	**4.87 (5.82)**	**0.086**
Number of positive nodes removed in cN0 cases [mean (SD)]	**0.36 (1.37)**	**0.95 (3.13)**	**0.30 (1.03)**	**0.062**
Axillary surgery in cN+ cases [*n* = 699]				**>0.9**
SLN	269/699 (38.5)	25/66 (37.9)	244/633 (38.5)	
ALND	430/699 (61.5)	41/66 (62.1)	389/633 (61.5)	
Mean total nodes removed in cN+ cases [mean (SD)]	11.82 (8.99)	11.58 (8.36)	11.84 (9.06)	>0.9
Mean positive nodes in cN+ cases [mean (SD)]	2.19 (4.22)	3.41 (5.62)	2.06 (4.03)	**0.024**

Data are expressed as *n*/*N* (%) unless otherwise specified

*SD* standard deviation, *BCS* breast-conserving surgery, *SLN* sentinel lymph node, *ALND* axillary lymph node dissection, *cN0* clinical node-negative, *cN+* clinical node-positive

^a^Wilcoxon rank-sum test, Fisher’s exact test, Pearson’s Chi-square test

Tumor margin status was available in 688/1329 patients (51.8%), including 67.7% of lobular cases (*n* = 84) and 50.1% of non-lobular cases (*n* = 604). Of these, positive margin rates varied by type of surgical procedure performed. For patients who underwent BCS as their initial surgery, positive margins were significantly more common in the ILC group compared with the non-ILC group (21.2% vs. 7.9%; *p* = 0.023). When mastectomy was performed as the initial surgery, the difference in positive margin rates by histologic subtype did not reach statistical significance (7.8% in lobular cases vs. 2.4% in non-lobular cases; *p* = 0.058) [Table 2].

Notably, Mammaprint class was associated with type of breast surgery overall, with a higher mastectomy rate in H1 tumors. Among patients with H2 tumors, 52.9% underwent mastectomy compared with 59.5% in the H1 cases (*p* = 0.016) [ESM Table S2].

### Axillary Surgery and Nodal Response

Overall, 805 patients (60.6%) underwent SLN surgery only and 524 patients (39.4%) underwent ALND (± SLN surgery). The mean number of nodes removed at SLN surgery was 3.34 (SD 2.27) and at ALND was 16.77 (SD 8.11). While patients in the lobular cohort had similar rates of cN+ disease at diagnosis as those in the non-lobular cohort, the rates of ypN+ disease at surgery differed significantly. Patients in the lobular cohort had a significantly higher proportion of ypN+ status compared with the non-lobular cohort (41.9% vs. 32.4%; *p* = 0.031).

When stratified by clinical nodal status, patients with ILC underwent different axillary management. Among cN0 patients, ALND was significantly more common in the lobular group than in the non-lobular group (24.1% vs. 14.0%; *p* = 0.039) [Table 2]. In these cN0 patients, there was no difference in the number of positive nodes found at surgery by histology (mean positive nodes 0.95 vs. 0.30 in lobular vs. non-lobular cases; *p* = 0.062). Among cN+ patients, the rates of ALND did not differ by histologic subtype (62.1% in the lobular cohort and 61.5% in the non-lobular cohort); however, those with lobular histology had more positive nodes at surgery than those with non-lobular histology (mean positive nodes of 3.41 vs. 2.06; *p* = 0.024).

**Fig. 3:**
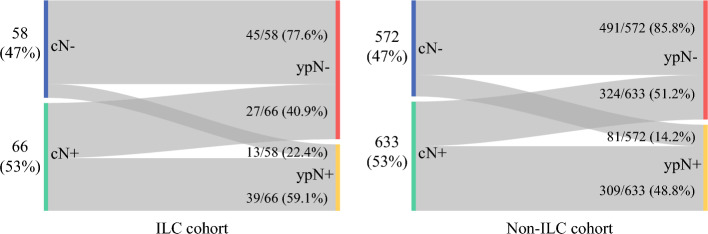
Sankey plots demonstrating the proportion of cN0 versus cN+ patients in each histologic group and how these relate to pathologic node status. Among cN+ patients in the ILC cohort, 40.9% converted to ypN-negative status after neoadjuvant chemotherapy; among cN+ patients in the non-ILC cohort, 51.2% converted to ypN-negative status. *cN0* clinical node-negative, *cN+* clinical node-positive, *ILC* invasive lobular carcinoma

In the entire study population, there were 630 cN0 patients (47.4%), of whom 85.1% were confirmed to be ypN0 at surgery, while the remaining 14.9% were found to be ypN+. Of the 699 cN+ patients, 49.8% remained ypN+ at surgery and the remaining 50.2% converted to ypN0 status. There was no difference in nodal conversion rates by histologic subtype, both overall and when stratified by tumor receptor subtype (ESM Table S3). Among cN0 patients, the proportion of ypN+ status at surgery was 22.4% in the lobular cohort and 14.2% in the non-lobular cohort (*p* = 0.093) [Fig. 3]. Among cN+ patients, the proportion of ypN0 status at surgery was 40.9% in the lobular cohort and 51.2% in the non-lobular cohort (*p* = 0.11) [Fig. 3].

**Fig. 4:**
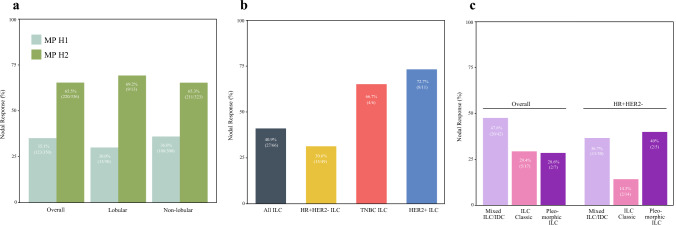
Bar graphs depicting nodal pCR rates in various subgroups. Nodal response is defined as the proportion of cN+ patients who converted to pathologic node-negative status (ypN0). (**a**) Higher rates of nodal pCR in MP H2 tumors. (**b**) Rates of nodal pCR among the ILC cohort stratified by tumor receptor subtype. (**c**) Rates of nodal pCR among ILC patients stratified by subtype, both overall and among HR+/HER2− cases. *pCR* pathologic complete response, *cN+* clinical node-positive, MP H2 Mammaprint high 2, *ILC* invasive lobular carcinoma, *HR* hormone receptor, *HER2* human epidermal growth factor receptor 2, *TNBC* triple-negative breast cancer, *IDC* invasive ductal carcinoma

MammaPrint class was significantly associated with both type of axillary surgery and conversion to ypN0 status among both the lobular and non-lobular cohorts (Fig. 4a). Overall, the rate of ALND was lower in patients with H2 tumors compared with H1 tumors (32.0% vs. 47.9%; *p* = 0.001) [ESM Table S4]. Additionally, the proportion of conversion from cN+ to ypN0 was 35.1% in H1 cases and 65.5% in H2 cases (*p* < 0.001). This was true within the lobular and non-lobular cohorts.

### Nodal Response Rates in Lobular Cases by Subtype

In the lobular cohort specifically, there were 66 cN+ patients, of whom 40.9% converted to ypN0 status. When stratified by tumor receptor subtype, the proportion of nodal conversion from cN+ status to ypN0 status was 30.6% in HR+/HER2−, 66.7% in triple-negative, and 72.7% in HER2+ lobular cases (Fig. 4b).

For patients with cN+ILC, we found a numeric difference in the proportion of conversions to node-negative status by ILC subtype but this did not reach statistical significance (Fig. 4c). Among HR+/HER2−ILC cases, the proportion of cN+ patients converting to ypN−status was 14.3% in classic ILC, 36.7% in mixed ILC/IDC, and 40.0% in pleomorphic ILC (Fig. 4c). Of the 49 lobular cases who converted from cN+ to ypN0, 15 (30.6%) had HR+/HER2−tumors, of which 11 were mixed ILC/IDC, 2 were classic, and 2 were pleomorphic. When comparing the HR+/HER2− ILC patients who converted to node-negative status after NAC versus those who remained ypN+, we did not find differences in age or tumor grade, but there appeared to be fewer premenopausal patients and more H2 tumors among those with nodal pCR (ESM Table S5).

Among the cN+HR+/HER2−lobular cases with Mammaprint H1/H2 class available (47/49), most tumors were H1 (*n* = 42, 89.4%), with only 5 cases being H2 (all mixed ILC/IDC). In these 5 H2 cases, 80% converted from cN+ to ypN0. In the 42 H1 cases, 6/24 mixed, 1/13 classic, and 2/5 pleomorphic cases converted from cN+ to ypN0 (ESM Table S6).

## Discussion

In this secondary data analysis of patients in the I-SPY2 trial, we found differences in surgical management by histologic subtype, and associations between nodal response based on both molecular and histologic features. While rates of mastectomy and BCS were similar in both lobular and non-lobular cases, patients with lobular tumors had significantly higher rates of positive margins after BCS. Additionally, among cN0 patients, lobular histology was associated with higher rates of ALND but no difference in the mean number of positive nodes at surgery. Finally, when we examined nodal response rates by histology, we interestingly found no significant difference in conversion from cN+ to ypN0 status between the lobular and non-lobular cohorts, suggesting that among Mammaprint high-risk tumors, overall nodal response rates to NAC appear similar regardless of tumor histology.

While others have shown higher rates of mastectomy in patients with lobular histology, we found no difference in mastectomy rates by histology in this study.^23^ This could reflect the inclusion of higher-stage tumors in the I-SPY2 trial as well as patient preference, or could reflect higher response rates in the primary tumor in patients with ILC. Although mastectomy rates were similar, we did find that patients with ILC had more positive margins after BCS, which has been shown previously. The positive margin rate of 21.2% in the lobular cohort in this study may be lower than expected for clinical stage II/III tumors as other groups have shown positive margin rates of up to 55% after NAC.^24–26^ This may reflect a greater degree of tumor downstaging in these molecularly selected, Mammaprint high-risk ILC cases, although we cannot conclude that definitively.

Regarding management of the axilla, prior investigators have shown that patients with ILC are more likely to present with nodal involvement and more likely to have nodal upstaging than patients with non-lobular tumors.^27^ Occult nodal disease in lobular breast cancer can be difficult to ascertain on imaging, particularly after neoadjuvant therapy where studies show that even breast MRI has relatively low accuracy for detecting nodal disease.^28^ This uncertainty may have driven surgical decisions about axillary management, reflected in the higher rates of ALND in cN0 patients with ILC in this study. It is also possible that de-escalation of axillary surgery was performed less often, again due to concerns about the ability to detect nodal metastases. Importantly, missing data on clinical assessment of the axilla after NAC is a limitation of this analysis.

Perhaps the most novel finding from this analysis is the relatively high rate of nodal conversion from cN+ to ypN0 status in the lobular cohort, with an overall nodal pCR rate of 40.9% in the lobular cohort (and 30.6% in the HR+/HER2− subset). Other studies that have evaluated nodal pCR in ILC have typically shown rates of < 15%, with higher rates seen in non-classic, triple-negative, or HER2+ ILC.^29–34^ The association between non-classic ILC and tumor receptor subtype can confound such data since a much higher proportion of non-classic ILC cases are either triple-negative or HER2+ subtypes that are known to have higher response rates to chemotherapy. Indeed, we also found this in our study, where the triple-negative and HER2+ lobular cases had very high rates of nodal pCR (66.7% and 72.7%, respectively), which was similar to non-lobular cases.

The more typical HR+/HER2− lobular cases remain the group in need of better stratification tools to predict chemotherapy response. We found a surprisingly high rate of nodal pCR in this group, occurring in 30.6% of patients. Because this study included only Mammaprint high-risk tumors, we surmise that the selection of these molecularly high-risk tumors identified a subset of lobular cases with a better chance of benefit from NAC.^21,35^ Within this group, we attempted to identify other factors associated with nodal pCR but were limited by the small sample size. Despite this, some notable observations include that the nodal pCR cases in the HR+/HER2−group were not limited to grade 3 cases, nor were they limited to the pleomorphic subtype. Within the HR+/HER2− lobular cases, Mammaprint class (H1 vs. H2) was significantly associated with nodal pCR, which is a useful finding for mixed ILC/IDC cases but less useful in classic ILC given the paucity of H2 cases. Indeed, we found that mixed ILC/IDC cases appeared to have the best chance of achieving nodal pCR. In our analysis, we chose to include mixed ILC/IDC cases in the lobular cohort as these cases have been shown to have clinical behavior akin to that of classic ILC.^36–38^

The high prevalence of mixed lobular/ductal cases in the lobular cohort suggests that these mixed cases may be more likely to have high genomic risk than classic cases. There is considerable uncertainty regarding this entity of mixed tumors.^39^ Prior studies indicate that this terminology may be variably used to describe tumors with intermixing of E-cadherin-negative and E-cadherin-positive cells, a collision tumor of a separate ILC and IDC, or tumors with a morphologically lobular growth pattern yet E-cadherin positivity.^40^ Additionally, the World Health Organization classification criteria for mixed invasive cancers have changed over time. Whereas the prior definition of a mixed tumor required at least 50% of the tumor to be of NST, the current definition of mixed histology allows for only 10% of the tumor to be NST.^40,41^ Combined with a lack of consensus on the diagnostic approach to lobular tumors, the variation in definitions of mixed ILC/IDC represents a challenge in the study of lobular breast cancer.

Prior studies have shown that the genomic profiles of these mixed tumors may be more IDC- or ILC-like, suggesting that further classification methods may be needed.^16^ The use of genomic testing to identify molecularly high-risk tumors in this study may have enriched the population for these mixed tumors and raises the possibility that some of these mixed ILC/IDC tumors might be more IDC-like than ILC-like since pure ILC tumors have low rates of molecular high-risk status. Further analysis of this subgroup is ongoing but it appears that even if the ILC component of these mixed tumors may result in more diffuse growth pattern, understanding the molecular subtype with a genomic assay may still aid with treatment selection.

More consistency in the diagnosis of lobular tumors may improve our understanding of this heterogeneous tumor type, as studies have shown high interobserver variability between pathologists.^42,43^ The definition of lobular histology has been debated, with some suggesting the diagnosis should be based on morphologic criteria alone and others suggesting the use of immunohistochemistry staining for E-cadherin and other associated proteins such as p-120.^44,45^ While study-site pathologists on I-SPY2 are breast specialists, the lack of central pathology review is a limitation of the current analysis. Another limitation includes the missing data on margin status in a high proportion of cases and the relatively small sample size of ILC, although this study may represent one of the larger cohorts of Mammaprint high-risk lobular tumors, given their rarity.

Our findings suggest that the application of genomic testing in the preoperative setting may help direct therapy to a neoadjuvant approach versus an upfront surgery approach, especially for HR+HER2− cases, where the potential benefit of cytotoxic chemotherapy can be less clear. While data evaluating this approach specifically in patients with ILC are scant, preoperative genomic assay testing of core needle biopsy samples has been shown to be feasible, with high concordance between test results from core needle biopsy specimens versus surgical specimens.^46,47^ Our findings are consistent with those of the MINT trial, in which patients with genomically high-risk tumors who underwent NAC had higher rates of nodal downstaging than those with genomically low-risk tumors.^35^ Additionally, the NBREaST II study showed that preoperative genomic testing reclassified 9% of tumors, and results were associated with response to therapy and long-term outcomes.^48^

## Conclusion

These data demonstrate the highest nodal pCR rates reported to date for patients with lobular breast cancers, across all tumor receptor subtypes. These data support the use of the 70-gene assay for selecting patients for NAC, especially in the setting of clinical nodal positivity. Additionally, these findings reflect the complex heterogeneity within lobular breast cancer and the need for improved stratification tools for this important and common tumor type.

## Supplementary Information

Below is the link to the electronic supplementary material.

Supplementary file1 (XLSX 22 kb)
